# The Mallet vs. Hand Pressure

**Published:** 1869-07

**Authors:** 


*The Mallet vs. Hand Pressure.*—From the Proceedings of the "Massachusetts Dental Society" we have the particulars of an interesting trial to determine the difference of density between mallet-force and hand-pressure.

The operators were Drs. Wetherbee and Salmon, and the trial was conducted in the presence of a committee, appointed for the purpose, consisting of Drs. McDougall, Codman and Whitechurch.

An ordinary steel draw-plate had been provided. It was placed on a block of wood the size of a man's head, weighing six pounds. One end of the plate was covered on one side with a piece of copper covering the hole to be filled, which was the centre hole at the end nearest the right hand; the large or funnel end of the hole was placed upward, and the work commenced.

Dr Salmon, who led off, "struck the first blow" at eleven minutes to eight. He had previously provided himself with three of his automatic mallets of the latest make, and which he stated were the same as he had just used for filling a crown cavity.

The foil used was No. 3, and the rolls were each made of half a sheet: Dr. McDougall annealed the foil. Dr. Codman acted as Secretary, while the others amused themselves in various ways, and the "rappings" were loud and "spirited" at the table where Dr. Salmon sat.

---

The number of blows on the first piece were not counted; the second piece received 130 blows; the next 180; from that number up to 275, as the circumference of the hole increased, which was the largest number counted. The cavity was filled more than full, rounded up, burnished, and filed off even with the plate; then finished at the bottom and top, and driven out of the plate. The time taken was one hour and eight minutes.

Dr. Wetherbee then took his turn. The number of thrusts or hand blows was rather more during most of the filling than the blows of the mallet, ranging from 171 to 318, as the highest number, to the half sheet, though the secretary observed that less hand blows were given than mallet blows for the final condensation. The time was a few minutes longer, being one hour and thirty-four minutes, which included some resting spells. The filling was then finished as before, driven out, and the difference of the two weighed.

The size of the hole and filling at its smallest diameter was No. 11 of the wire gauge. The amount of foil used was a trifle over ⅛ oz.

The weight of the fillings finished was—Dr. Salmon's with the automatic mallet, 28 grains; Dr. Wetherbee's with the hand pressure, 24 grains; difference in weight 4 grains.

The committee believe the trial to have been conducted with perfect fairness in all particulars. The blows and pressure were heavy and greater than is usually used in filling teeth, and, as the committee believe, more than is generally practicable. If any advantage existed, they think it was in favor of the mallet operator, as, the hand pressure being exerted in a different position from usual, the instrument rested in an unusual position, and chafed the hand of the operator, making it sore even to blistering. We estimate that nearly or quite five thousand blows were given to each filling.

